# Targeted repression of *Plasmodium* apicortin by host microRNA impairs malaria parasite growth and invasion

**DOI:** 10.1242/dmm.042820

**Published:** 2020-06-03

**Authors:** Malabika Chakrabarti, Swati Garg, Ayana Rajagopal, Soumya Pati, Shailja Singh

**Affiliations:** 1 Host Parasite Interactions and Disease Modeling Lab, Special Centre for Molecular Medicine, Jawaharlal Nehru University, New Delhi 110067, India; 2 Animal Physiology and Neurobiology, Katholieke Universiteit Leuven, Naamsestraat 59 - Box 2465, Belgium; 3 Department of Life Sciences, School of Natural Sciences, Shiv Nadar University, Gautam Budh Nagar, Noida, UP 201314, India

**Keywords:** miR-150-3p, miR-197-5p, Apicortin, Hybridization, Microneme secretion, AMA1, Malaria, Invasion, *Plasmodium*, Erythrocytes

## Abstract

Mature human erythrocytes contain a rich pool of microRNAs (miRNAs), which result from differentiation of the erythrocytes during the course of haematopoiesis. Recent studies have described the effect of erythrocytic miRNAs on the invasion and growth of the malaria parasite *Plasmodium falciparum* during the asexual blood stage of its life cycle. In this work, we have identified two erythrocytic miRNAs, miR-150-3p and miR-197-5p, that show favourable *in silico* hybridization with *Plasmodium* apicortin, a protein with putative microtubule-stabilizing properties. Co-expression of *P. falciparum* apicortin and these two miRNAs in a cell line model resulted in downregulation of apicortin at both the RNA and protein level. To create a disease model of erythrocytes containing miRNAs, chemically synthesized mimics of miR-150-3p and miR-197-5p were loaded into erythrocytes and subsequently used for invasion by the parasite. Growth of the parasite was hindered in miRNA-loaded erythrocytes, followed by impaired invasion; micronemal secretion was also reduced, especially in the case of miR-197-5p. Apicortin expression was found to be reduced in miRNA-loaded erythrocytes. To interpret the effect of downregulation of apicortin on parasite invasion to host erythrocytes, we investigated the secretion of the invasion-related microneme protein apical membrane antigen 1 (AMA1). AMA1 secretion was found to be reduced in miRNA-treated parasites. Overall, this study identifies apicortin as a novel target within the malaria parasite and establishes miR-197-5p as its miRNA inhibitor. This miRNA represents an unconventional nucleotide-based therapeutic and provides a new host factor-inspired strategy for the design of antimalarial molecular medicine.

This article has an associated First Person interview with the first author of the paper.

## Abstract (precis)

**Summary:** The role of host erythrocyte microRNA in the downregulation of malaria parasite gene expression is investigated. Two microRNAs are identified, miR-197-5p and miR-150-3p, which affect parasite growth and invasion when enriched in erythrocytes.

## INTRODUCTION

Malaria remains the primary cause of morbidity and mortality for a large segment of the population worldwide (Cowman et al., 2016). According to the World Malaria Report 2018, approximately 219 million cases of malaria infection were reported across 90 countries, leading to 435,000 deaths (World Health Organization, 2019). The asexual blood stage of malaria parasites, *Plasmodium* spp*.*, attributes to the clinical symptoms and pathology of the disease (Church et al., 2003; Trampuz et al., 2003; de Koning-Ward et al., 2016; Tangpukdee et al., 2009). Thus, targeting parasite proteins that have crucial roles in intra-erythrocytic growth remains a key focus in the development of curative therapeutics against malaria (De Beer et al., 2009; Muregi et al., 2011; Gelb, 2007). The development of resistance against all known drugs by the malaria parasite has necessitated the design of innovative therapeutics with novel modes of action (World Health Organization, 2019). In this regard, host-inspired therapeutics are being explored to overcome the problem of drug resistance (Sidhaye et al., 2016; Uneke, 2007).

Different erythrocyte phenotypes have an important role as host factors affecting parasite biology; for example, absence of the erythrocyte Duffy receptor protein reduces *Plasmodium* *vivax* infection (Mueller et al., 2009; Maier et al., 2003). Similarly, erythrocytes exhibiting the Gerbich-negative phenotype show reduced infection by *Plasmodium* *falciparum*, owing to a mutation in exon 3 of the gene encoding glycophorin C, which is crucial for invasion together with erythrocyte-binding antigen 140 (EBA-140) (Rowe et al., 2009; Imrie et al., 2007). Ovalocytosis and reduced expression of complement receptor 1 have also been linked to impaired invasion (Min-Oo and Gros, 2005). Likewise, altered expression of host erythrocyte cytosolic enzymes can lead to reduced intracellular growth of the malaria parasite: attenuated expression of glucose-6-phosphate dehydrogenase and pyruvate kinase results in the efficient phagocytosis of infected erythrocytes (Zanella et al., 2005; May et al., 2000; Allison, 2009; Roberts and Williams, 2003). Erythrocytes with altered structural variants of haemoglobin (HbS, HbC and HbE) or the abnormal form of haemoglobin found in thalassemia show reduced invasion, growth and egress of the parasites (Taylor et al., 2012; Chotivanich et al., 2002; Ayi et al., 2004; Lell et al., 1999; Rathjen et al., 2006). In addition to these factors, recent studies have identified microRNAs (miRNAs) as crucial host factors that regulate parasite growth (Rubio et al., 2016; Chang and Mendell, 2007). MicroRNAs are endogenous, non-coding nucleotides of 20-22 bp length that participate in different important physiological processes (e.g. cell proliferation, differentiation and neuronal development), as well as having a role in diverse pathological processes such as host pathogen interaction, inflammation, apoptosis and tumorigenesis (Havelange and Garzon, 2010; Singh et al., 2013; Merkerova et al., 2008). Transcriptomic studies have revealed the presence of a diverse pool of miRNAs in the developmental and mature stages of erythrocytes. These miRNAs have a key role in haematopoiesis and maturation of different lineages of blood cells, including erythrocytes (Ryan and Atreya, 2011; Kannan and Atreya, 2010; Teruel-Montoya et al., 2014; Juzenas et al., 2017; Chen et al., 2008). Moreover, microvesicles released from erythrocytes have been found to contain miRNA coupled with Argonaute 2, which can lead to altered gene expression in other types of cells (e.g. barrier function in brain endothelial cells) (Mantel and Marti, 2014; Mantel et al., 2016). Also, erythrocytes of the sickle cell phenotype have been found to be enriched with specific miRNAs that lead to growth inhibition of the malaria parasite through the translational repression of parasite mRNA (LaMonte et al., 2012). Further investigation in this area could lead to the development of host physiology inspired novel therapeutics based on miRNAs.

The work presented here reports a novel host-inspired miRNA-mediated therapy to restrict the growth of intra-erythrocytic parasites. Briefly, two candidate miRNAs (miR-150-3p and miR-197-5p) were found to hybridize favourably with the gene encoding apicortin (a member of the apicomplexan family of genes) through *in silico* hybridization of erythrocytic miRNAs against the *P. falciparum* and *P. vivax* transcriptome. The anti-apicortin activity of the selected miRNAs was confirmed in cell line and erythrocyte models enriched with miRNA mimics. Translational repression of *P. falciparum* apicortin (PfApicortin) led to reduced parasite growth and attenuated merozoite invasion. Moreover, downregulation of PfApicortin in merozoites led to reduced micronemal discharge. We propose that the antimalarial activity displayed by human miR-197 could be further developed as a next-generation antimalarial molecular medicine.

## RESULTS

### miR-150-3p and miR-197-5p hybridize with *Plasmodium* apicortin

**Fig. 1.**
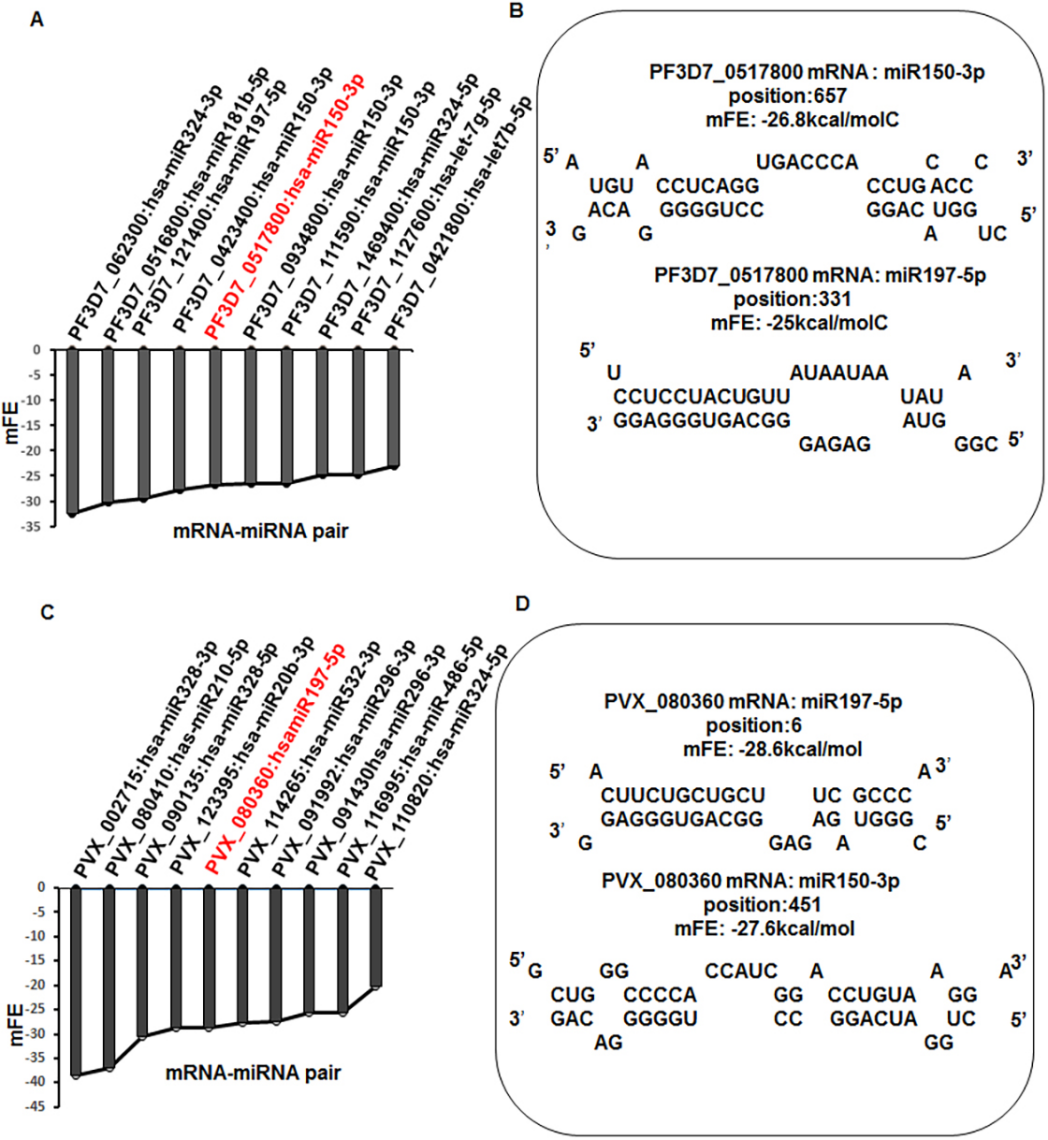
**miR-150-3p and miR-197-5p hybridize with *Plasmodium* apicortin.** (A) Graph showing ten miRNA-mRNA pairs starting from the pair with the lowest energy of binding in the case of *P. falciparum*; the pair formed between PfApicortin and miR-150 is highlighted in red. (B) Mode of hybridization of PfApicortin mRNA with miR-150 and miR-197 showing the location within the mRNA where binding occurs. (C) Graph showing ten miRNA-mRNA pairs starting from the pair with the lowest energy of binding in the case of *P. vivax*; the pair formed between *P. vivax* apicortin and miR-197 is highlighted in red. (D) Mode of hybridization of *P. vivax* apicortin mRNA with miR-150 and miR-197 showing the location within the mRNA where binding occurs.

*In silico* hybridization data indicate energetically favourable binding of erythrocyte miRNAs with malaria parasite genes (Fig. 1, Table S1). Apicortin, a protein with a putative microtubule stabilization function, appeared to be one of the targets of hybridization. In the case of *P. falciparum*, the best hybridization fit was observed with miR-150-3p (Fig. 1A), whereas for *P. vivax* it was miR-197-5p (Fig. 1C). The minimum free energy of binding of miR-150-3p with *P. falciparum* apicortin was −26.8 kcal/mol and the site of seed sequence binding was located 359 bp from the 3′ end of the RNA (Fig. 1B). Similarly, the minimum free energy of binding of miR-197-5p with *P. vivax* apicortin was −28.6 kcal/mol and the site of seed sequence binding was located 23 bp from the 5′ end of the RNA (Fig. 1D). To check the species specificity of the two hybridizing miRNAs, miR-150-3p was hybridized with *P. vivax* apicortin and miR-197-5p was hybridized with *P. falciparum* apicortin. No significant change in hybridization energy was observed in either case (Fig. 1B,D). Hybridization of miRNA seed sequences with the target gene indicated effective assembly of an mRNA-miRNA pair. The data also show that the candidate miRNAs are not species specific. Thus, the miRNA-mediated downregulation of PfApicortin might have a possible role in the destabilization of parasite microtubule assembly.

### Downregulation of PfApicortin transexpressed in HEK 293T cells by miR-150 and miR-197

**Fig. 2.**
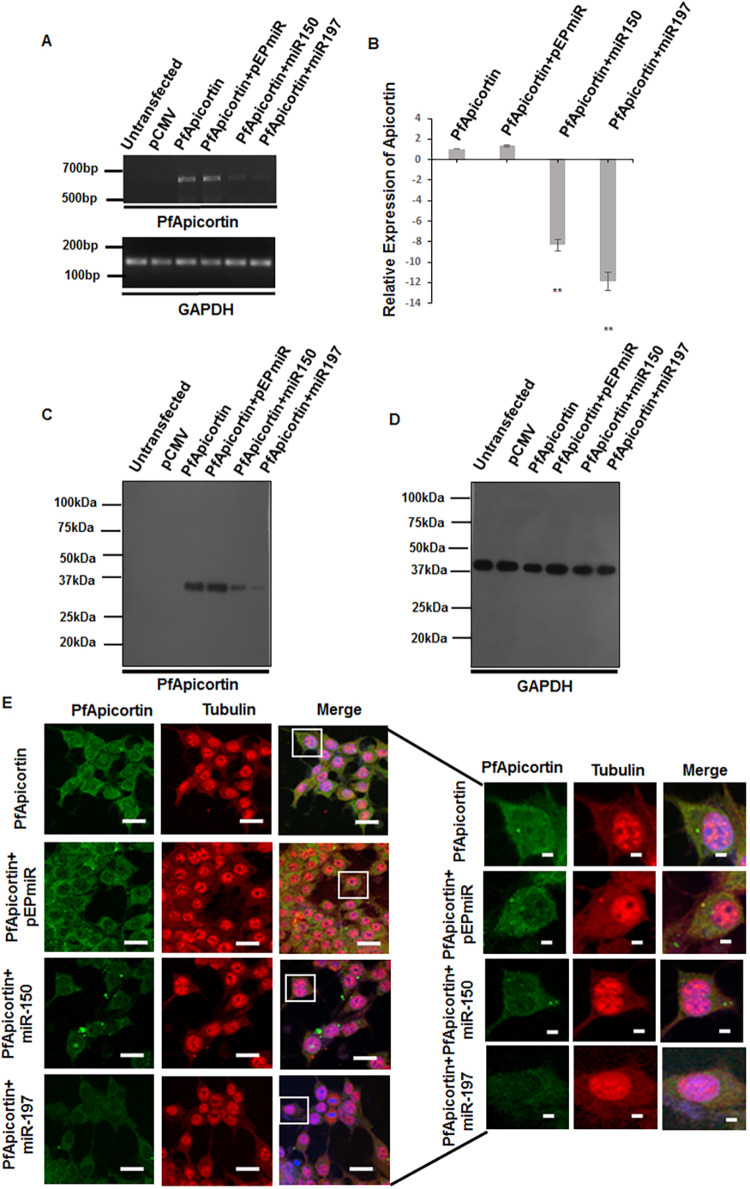
**Downregulation of *P. falciparum* apicortin trans-expressed in HEK 293T cells by miR-150 and miR-197.** (A) Semiquantitative PCR showing reduced intensity of the PfApicortin band (645 bp) in miRNA-transfected cells (full gel image shown in Fig. S2A). (B) qPCR data showing downregulation of PfApicortin by miR-150 (**P<0.01) and miR-197 (**P<0.01). (C) Western blot showing reduced expression of apicortin in miRNA-cotransfected cells; significant downregulation of PfApicortin expression by miR-150 (*P*<0.01) and miR-197 (*P*<0.001) over control was observed (graph shown in Fig. S2C). (D) Western blot showing GAPDH as loading control. (E) Immunofluorescence assay images of transfected cells with PfApicortin in the green channel and tubulin (as control) in the red channel; individual cells are shown in magnified form. PfApicortin expression was reduced in cells transfected with miR-150 (*P*<0.001) or miR-197 (*P*<0.001, graph shown in Fig. S2D). Nuclei were counterstained with DAPI. Data expressed as mean±s.d. from three independent experiments. Scale bars: 5 µm.

To replicate *in silico* data, PfApicortin (cloned in pCMV, Fig. S1A, Table S2), pre-miR-150 and pre-miR-197 (cloned in pEP-Mir, Fig. S1B,C) were co-transfected in HEK 293T cells. Cells transfected with PfApicortin alone or PfApicortin together with the pEP-Mir empty vector were used as controls. PfApicortin expression was checked at 48 h after transfection through semiquantitative and real-time PCR followed by western blotting and immunofluorescence assay. Semiquantitative PCR demonstrated reduced band intensity for PfApicortin in miRNA-cotransfected samples (Fig. 2A, Fig. S2A,B). Further confirmation was achieved by real-time PCR of transfected cell cDNA, which showed 8-fold and 11.7-fold downregulation of PfApicortin by miR-150 and miR-197, respectively (Fig. 2B). Expression of PfApicortin was also monitored by immunoblotting of cell lysate after 48 h transfection: a significant downregulation (miR150, *P*<0.01; miR197, *P*<0.001) of PfApicortin was observed in miRNA-transfected samples, compared with controls (Fig. 2C, Fig. S2C). GAPDH was used as a loading control (Fig. 2D). Immunofluorescence assays also showed a significant reduction of fluorescence intensity of PfApicortin (*P*<0.001) in miRNA-cotransfected cells compared with controls (Fig. 2E, Fig. S2D).

### Expression of PfApicortin in the asexual blood stage of malaria parasite

**Fig. 3.**
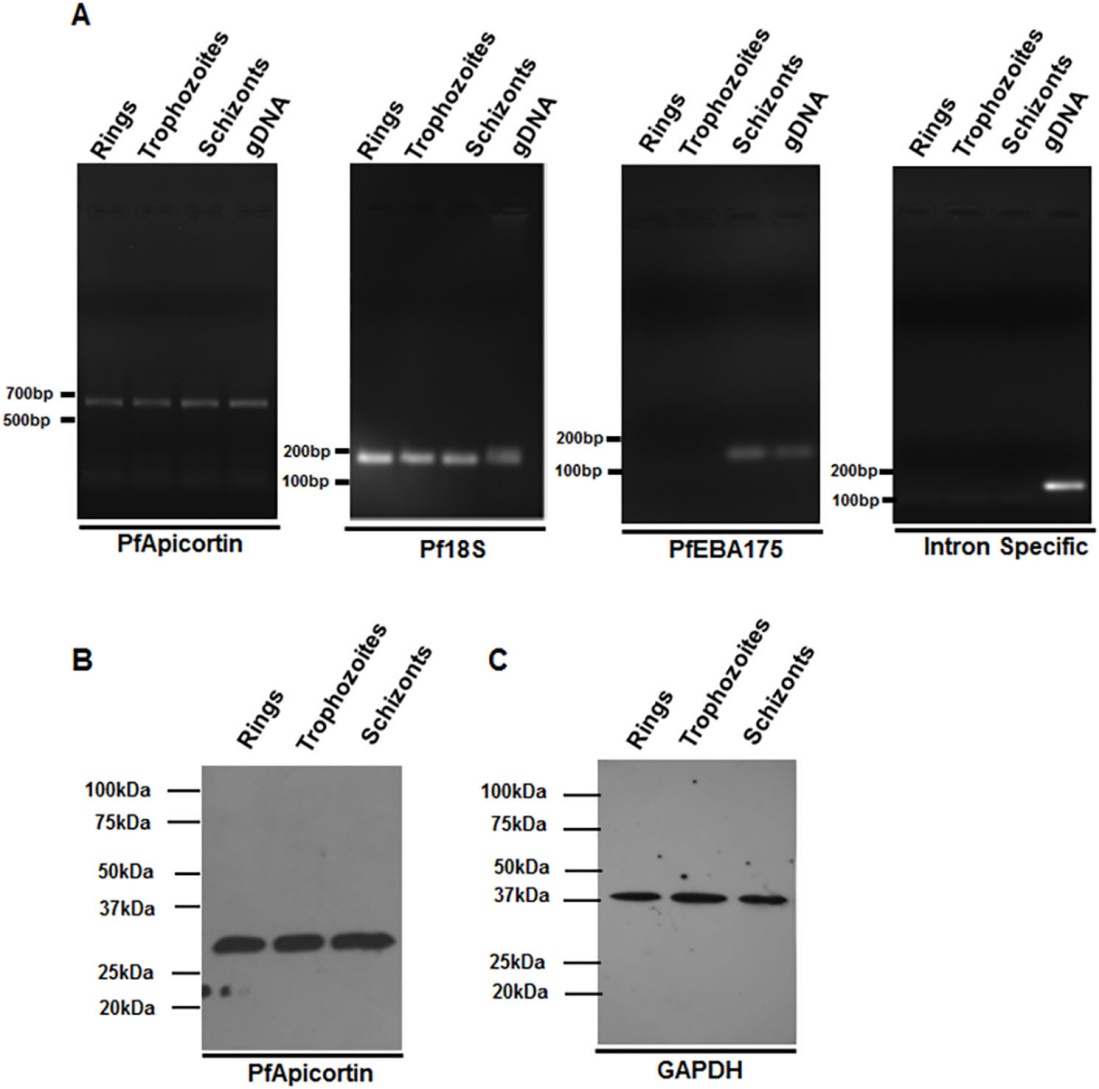
**Expression of PfApicortin in the asexual blood stage of malaria parasite.** (A) Semiquantitative PCR of PfApicortin (645 bp), Pf18S (150 bp), PfEBA-175 (125 bp) and an intron-specific region (140 bp) using ring, trophozoite and schizont cDNA and genomic DNA of *P. falciparum* 3D7. (B) Western blot of PfApicortin showing its expression in the ring, trophozoite and schizont stages with a band at 28 kDa. (C) Western blot showing GAPDH as loading control. Data expressed as mean±s.d. from three independent experiments.

To monitor expression of PfApicortin at the transcription level, semiquantitative PCR was performed using cDNA from the ring, trophozoite and schizont stages as template. Bands of equal intensity were observed at 645 bp for all stages, indicating a constant level of expression of PfApicortin (Fig. 3A). Primers against Pf18S, PfEBA-175 and an intron-specific region were used as controls (Fig. 3A). Expression of PfApicortin was also monitored at the protein level by immunoblotting lysate from the ring, trophozoite and schizont stages with anti-PfApicortin antibody. A band of ∼28 kDa was observed in all stages, confirming the steady expression of PfApicortin throughout the blood stage (Fig. 3B). GAPDH was used as loading control in this experiment (Fig. 3C).

### Localization of PfApicortin in the asexual blood stage of malaria parasite

**Fig. 4.**
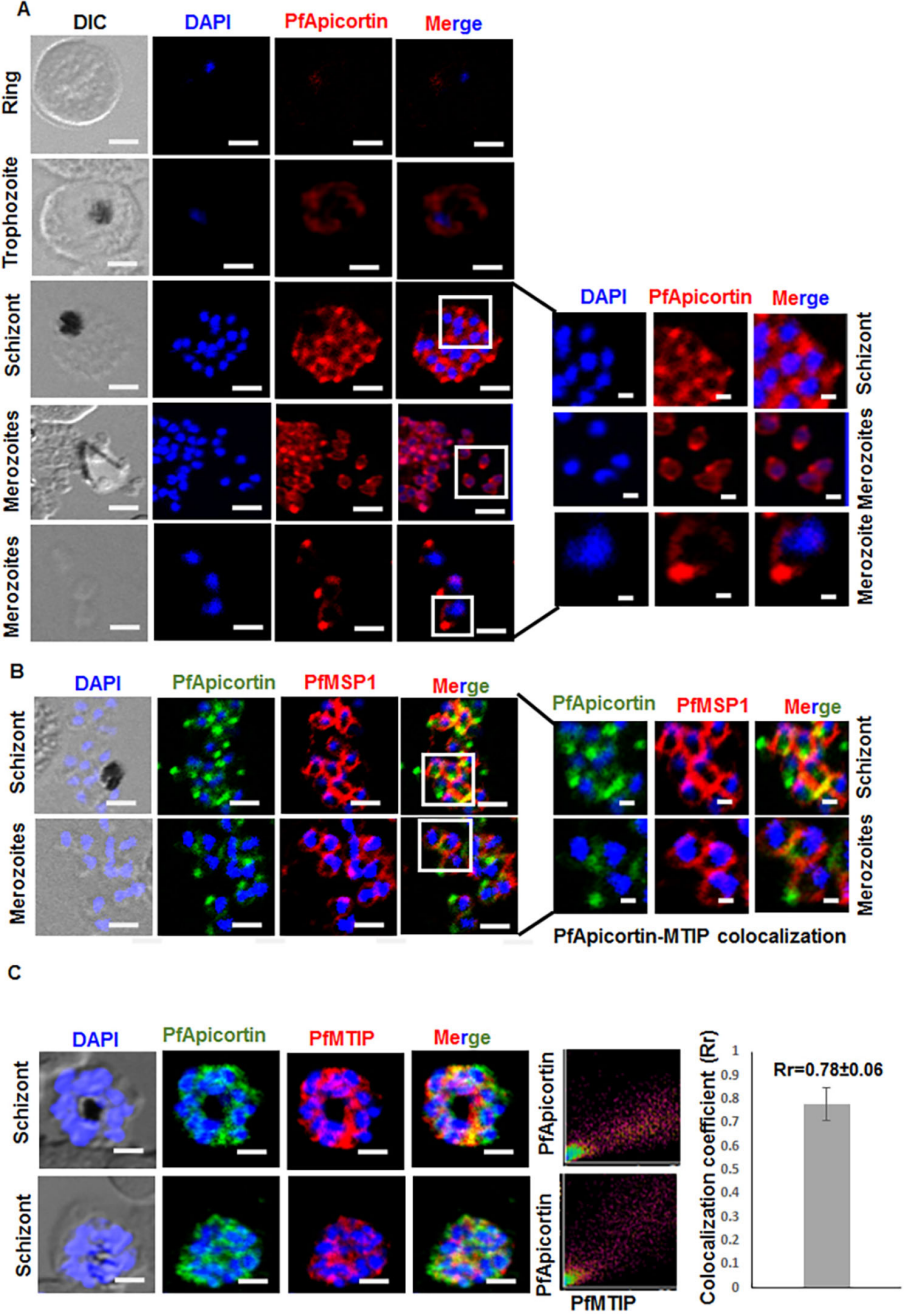
**Localization of PfApicortin in the asexual blood stage of malaria parasite.** (A) Localization of PfApicortin in ring, trophozoite, schizont and merozoite stages. Apicortin is shown in red with magnified images of the schizont and merozoite stages. (B) The presence of PfApicortin in schizont and merozoite stages together with MSP1 as control with magnified images of a portion of the schizont and some of the merozoites. Nuclei were counterstained with DAPI. (C) Colocalization of PfApicortin with MTIP in the schizont stage; the graph shows the colocalization coefficient. Data expressed as mean±s.d. from three independent experiments Scale bars: 5 µm.

We next attempted to study the localization of PfApicortin within the parasite. Immunofluorescence assay was performed using anti-PfApicortin antibody in methanol-fixed parasites of different stages. Expression of PfApicortin was observed in all stages. In merozoites, PfApicortin was localized to the apical end of the parasites (Fig. 4A). Localization of PfApicortin was further assessed in schizonts together with merozoite surface protein 1 (MSP1) using immunofluorescence assay (Fig. 4B). A study of PfApicortin and PfMTIP in mature schizonts demonstrated a high extent of colocalization (Fig. 4C, Pearson coefficient 0.78±0.06), indicating a possible role of PfApicortin in microtubule dynamics and invasion.

### Downregulation of PfApicortin and growth inhibition of parasites in miRNA-loaded erythrocytes

**Fig. 5.**
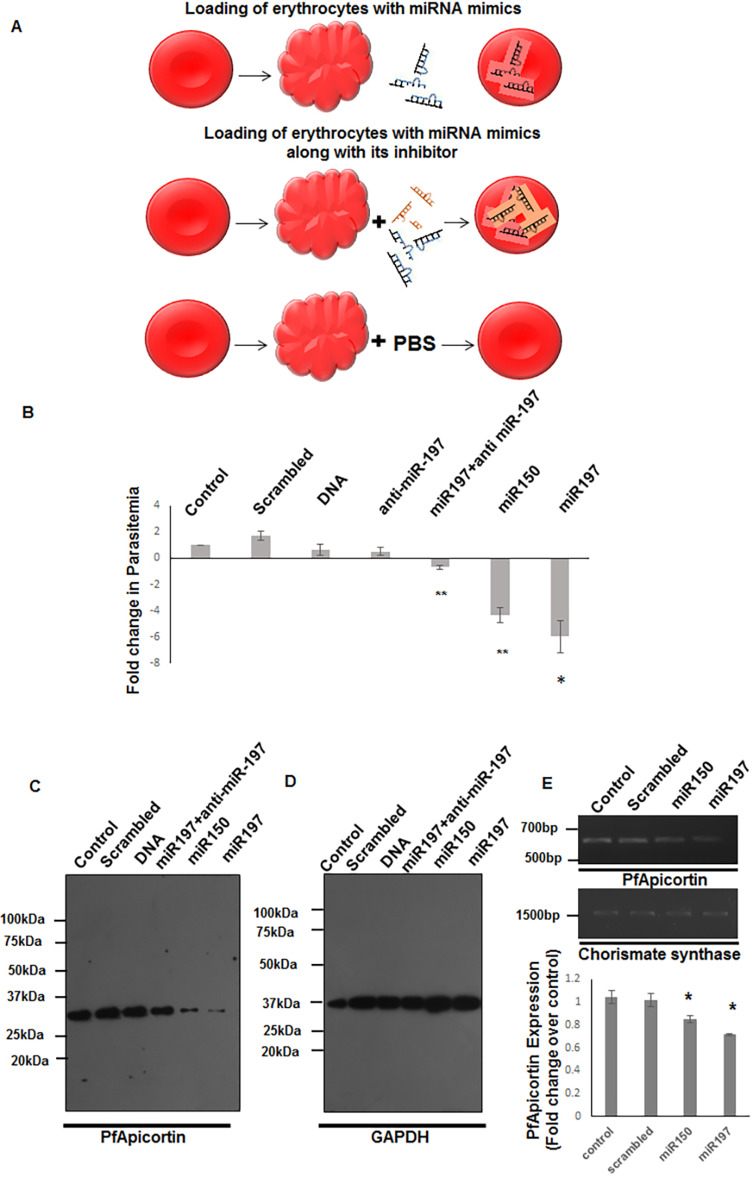
**Loading erythrocytes with miRNA mimics leads to growth inhibition of parasites and downregulation of PfApicortin.** (A) Schematic showing loading of erythrocytes with miRNA mimics or mock control. (B) Graph showing the reduction in growth (miR-150, **P<0.01; miR-197, *P<0.05; miR-197+anti-miR-197, **P<0.01) of parasites at 48 hpi in miRNA-loaded erythrocytes. (C) Western blot showing downregulation of PfApicortin by miR-150 and miR-197 (*P*<0.01, Fig. S3A). (D) Western blot showing GAPDH as loading control. (E) Semiquantitative PCR showing expression of PfApicortin and chorismate synthase in parasite-infected mimic-enriched erythrocytes (*P<0.05). Data expressed as mean±s.d. from three independent experiments.

To develop an erythrocyte model enriched with miRNAs, packed erythrocytes were loaded with mimics of miR-150-3p and miR-197-5p using an erythrocyte lysis and resealing method (Fig. 5A) (Chandramohanadas et al., 2009). Loaded erythrocytes were infected with *P. falciparum* 3D7 and its growth was monitored in comparison with infected erythrocytes loaded with scrambled miRNA mimic, PfApicortin PCR product, anti-miR-197-5p and mock control (erythrocytes lysed and resealed without any cargo). Parasite growth/progression was observed at different time points by monitoring Giemsa-stained smears. Growth of the parasites was significantly reduced (miR-150, *P*<0.01; miR-197, *P*<0.05) in erythrocytes loaded with the miRNA mimic: there was a 4- to 5-fold reduction in parasitemia 48 h after invasion (Fig. 5B). In the case of erythrocytes loaded with miR-197-5p together with its inhibitory molecule anti-miR-197-5p, growth reduction was less (*P*<0.01) suggesting effective blocking of miR-197 activity. In order to monitor the effect of miRNA mimics on PfApicortin expression, parasite cDNA was probed with PfApicortin primers using semiquantitative PCR. Parasites infecting erythrocytes loaded with the miRNA mimic showed a significant reduction in PfApicortin expression (Fig. 5E, Fig. S3C, *P*<0.05). The level of a non-target gene (chorismate synthase) was also monitored, to rule out the possibility of any non-specific effects. The level of chorismate synthase was equal in all cases (Fig. 5E, Fig. S3D). Mature schizonts of 40-42 h post-invasion (hpi) were incubated with miR-150 or miR-197 mimics at a concentration of 10 µM and expression was checked by immunoblotting of the merozoite pellet. PfApicortin PCR product (2 μg) and anti-miR-197-5p (10 μM) were used as negative controls along with scrambled miRNA mimic (10 μM). A significant reduction in the level of PfApicortin (*P*<0.01, Fig. 5C, Fig. S3A) was observed. GAPDH was used as loading control (Fig. 5D).

### Impaired invasion of parasites and reduced micronemal secretion owing to exposure to miRNA mimics

**Fig. 6.**
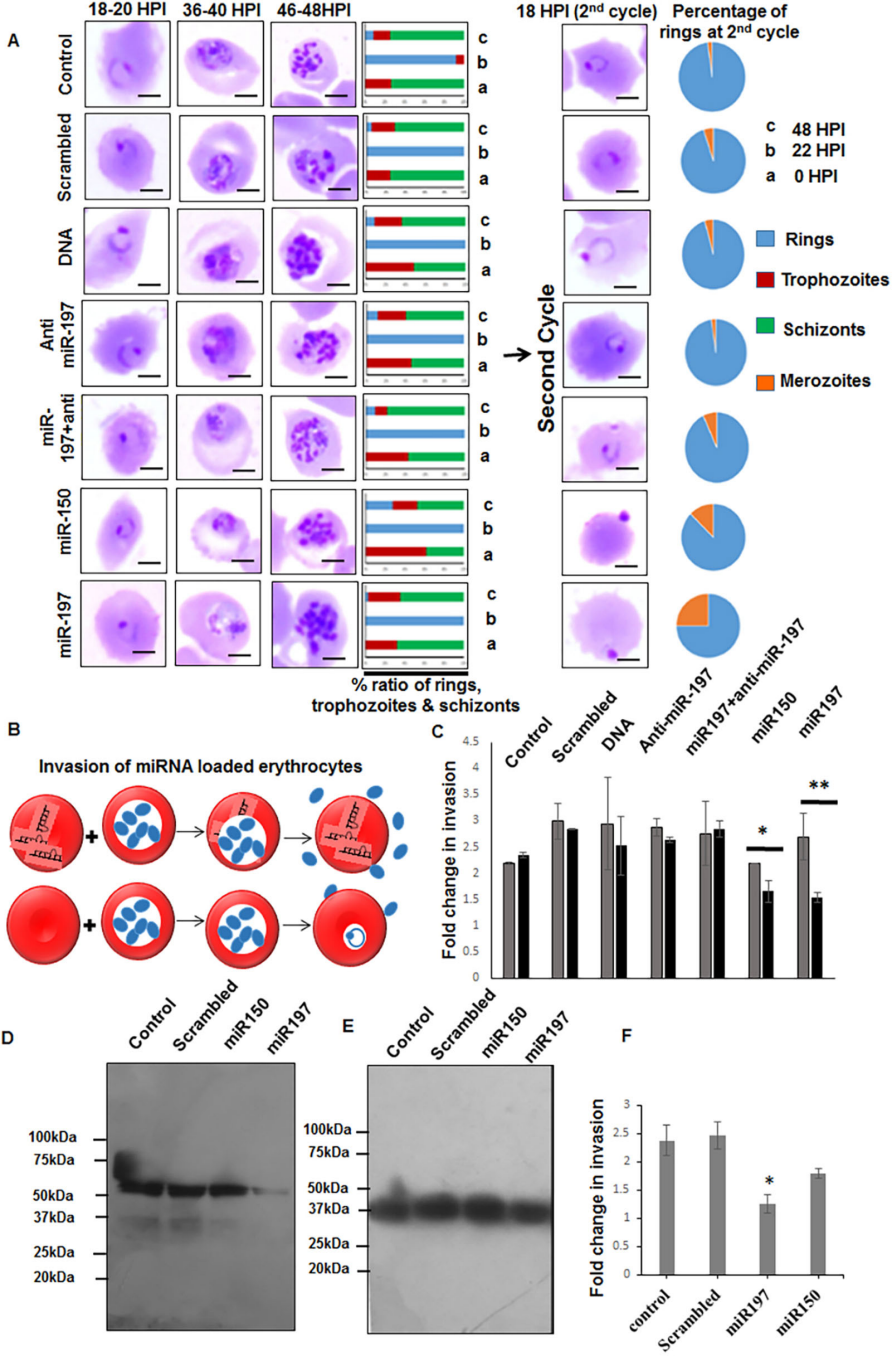
**Impaired invasion of parasites and reduced micronemal secretion owing to exposure to miRNA mimics.** (A) Progression of the parasites was monitored in miRNA-loaded and control erythrocytes. Images of Giemsa-stained parasites at 24 h, 36 h and 48 h demonstrate no significant change in parasite progression. The bar graphs demonstrate the ratio of rings, trophozoites and schizonts. Ring formation was accessed 18 hpi of the second cycle. Pie charts demonstrate the distribution of rings and invading merozoites in the second cycle of invasion. (B) Schematic showing invasion of parasites in miRNA-loaded erythrocytes. (C) Fold change in invasion by parasites in miRNA-loaded erythrocytes in two consecutive cycles of infection at 0 hpi and 72 hpi; grey and black bars show fold change in invasion at 0 hpi and 54 hpi, respectively. There is a significant reduction in invasion in miR-loaded erythrocytes (miR150, *P<0.05; miR197, **P<0.01) in the second cycle. (D) Western blot showing reduced secretion of AMA1 in merozoite culture supernatant pretreated with miR-150 and miR-197 (*P*<0.01, Fig. S3B). (E) Western blot showing GAPDH as loading control in the merozoite pellet. (F) Graph showing a significant reduction in invasion by merozoites pretreated with miR-197 (*P<0.05). Data expressed as mean±s.d. from three independent experiments.

Growth progression of malaria parasite was monitored in erythrocytes loaded with miRNA mimics. We found that the parasite progressed similarly in both control and treated erythrocytes; however, erythrocytes loaded with miR-150 and miR-197 mimics demonstrated a significant reduction in invasion in the second cycle, 18-20 h post-invasion. Furthermore, we found that a number of merozoites were attached to erythrocytes, but could not invade (Fig. 6A,B). The invasion of merozoites decreased significantly (miR-150, *P*<0.05; miR-197, *P*<0.01) in the second cycle, compared with the first cycle (Fig. 6C). In the case of erythrocytes loaded with scrambled miRNA mimic, anti-miR-197 or DNA, the invasion of parasites remained unaffected in the second cycle (Fig. 6A,C). To further confirm the effect of miRNA mimics on parasite invasion, mature schizonts (40-42 h old) were incubated with miRNA mimics and micronemal secretion was monitored in the lysate of merozoites obtained from the treated parasites. The level of apical membrane antigen 1 (AMA1) secretion was reduced significantly (*P*<0.01) in the merozoite culture supernatant obtained from miR-197-treated schizonts, compared with the mock control, scrambled mimic and miR-150 (Fig. 6D, Fig. S3B). GAPDH was used as loading control (Fig. 6E). Schizonts pretreated with miR-197 and miR-150 also demonstrated reduced invasion (Fig. 6F).

### Confirmation of the translocation of miR-150-3p and miR-197-5p in parasites infecting mimic-loaded erythrocytes

**Fig. 7.**
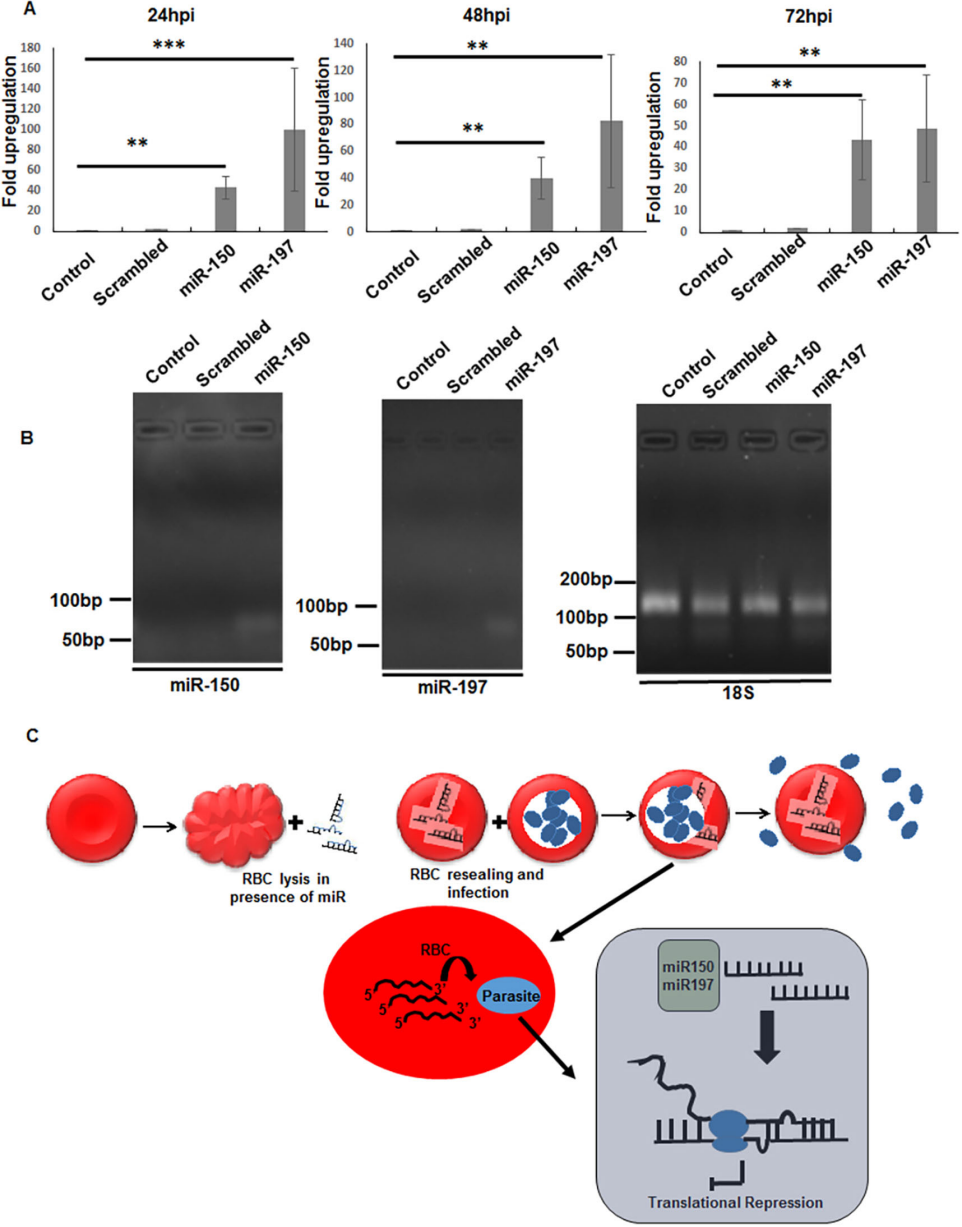
**Translocation of miR-150 and miR-197 at 24 hpi, 48 hpi and 72 hpi.** (A) Real-time PCR data showing fold upregulation of miR-150 and miR-197 over control at 24 hpi, 48 hpi and 72 hpi (miR-150, **P<0.01; miR-197, ***P<0.001 at t=24 hpi and **P<0.01 at t=48 hpi and 72 hpi). (B) Agarose gel images showing bands of miR-150, miR-197 and Pf18S at 48 hpi. Data expressed as mean±s.d. from three independent experiments. (C) Schematic showing development of an erythrocyte model enriched with miRNA and possible mechanisms of miRNA-mediated downregulation of parasite gene expression. Erythrocyte miRNA might affect gene expression through translocation to the parasite, as mentioned in previous studies. Complementarity with the seed sequence of miRNAs might lead to hybridization-mediated regulation of gene expression. The miRNA might bind with the parasite mRNA and simply induce translational repression by stalling ribosomal assembly through formation of a mRNA-miRNA hybrid. The mechanism supports host miRNA-mediated parasite gene expression and highlights strategies to design molecules inspired by the host defense mechanism. RBC, red blood cell.

To monitor the translocation of miRNA mimics to the parasite from loaded erythrocytes, polyadenylated parasite cDNA was probed with primers specific for miR-150-3p and miR-197-5p using real-time PCR at 24 h, 48 h and 72 h after invasion. Significant fold changes in miRNA levels were observed at different time points, compared with the infected erythrocyte control (without any cargo) or scrambled mimic enriched erythrocytes (Fig. 7A). The level of the specific miRNAs in the parasite during set up of the assay (t=0) was nil. Bands of miR-150-3p and miR-197-5p were observed at 55 bp upon running the PCR products in a 2% agarose gel (Fig. 7B). Pf18S was used as housekeeping control in the experiment (Fig. 7B). The primers (Table S2) showed specificity to their respective miRNA, as no signal or band was detected by miR-150 primers in cDNA samples containing miR-197 or vice versa (Fig. S3E,F).

## DISCUSSION

MicroRNAs are known to regulate a number of important biological processes in eukaryotes. Previous studies have revealed the presence of a rich pool of miRNAs in mature erythrocytes, leading to altered physiology of the cells and apoptosis (Juzenas et al., 2017). The population of erythrocytic miRNA might have a role in the regulation of different malaria parasite genes, affecting growth and invasion of the parasite during the asexual blood stage; the disease symptoms of malaria are also attributable to the asexual blood stage. Earlier studies have proven this hypothesis by linking different haemoglobinopathies, such as beta thalassemia or sickle cell anaemia, with the acquisition of resistance to parasite invasion and growth. An abundance of certain miRNAs, such as miR-451, let-7i and miR-223, in erythrocytes containing HbAS and HbSS was found to hinder parasite growth and invasion through downregulation of the cAMP-dependent protein kinase regulatory subunit (PKA-R). Downregulation of PKA-R is achieved through the process of trans-splicing, which occurs between the miRNA and 5′ end of the parasite cDNA and inhibits loading of the ribosomal complex onto the parasite transcript (LaMonte et al., 2012). The possible reason for trans-splicing is the homology between the erythrocytic miRNA sequence and the parasite expressed sequence tag. In this study, we have tried to further elaborate the mechanism of host miRNA-mediated parasite gene downregulation by using the natural process of miRNA-mediated regulation (i.e. hybridization of miRNAs with parasite transcripts), which has also been investigated in other recent studies (Wang et al., 2017; Dandewad et al., 2019).

Hybridization of erythrocytic miRNAs against parasite transcripts raises a question about the region of the mRNA to be hybridized against the miRNA. The absence of defined UTR regions in malaria parasite transcripts adds to the difficulty of hybridization. Binding of miRNA to the 3′-UTR is the commonest phenomenon, although in some cases binding occurs to the 5′-UTR. In humans, however, there is evidence of miRNA-mediated gene regulation through binding with the protein-coding region of the gene; this mechanism of regulation is also quite prevalent in plants (Duursma et al., 2008). Therefore, on the basis of earlier evidence, we hybridized erythrocytic miRNAs against the whole mRNA (including UTRs where available) of the parasite genes expressed in the trophozoite and schizont stages, as all the proteins crucial for parasite invasion and egress are expressed in these two stages. Apicortin was selected for target validation in this study, because of its unique presence in apicomplexan parasites together with its possible role in the stabilization of microtubule assembly (Orosz, 2009, 2011).

Co-expression of miRNA and its target in cell lines is a common strategy to validate the *in silico* hybridization data and to determine the efficiency of miRNA-mediated inhibition of gene expression. Reduction in the expression of PfApicortin by both miR-150 and miR-197 (Fig. 2) is a preliminary confirmation of the *in silico* data, as it does not specify the exact arm of miRNA responsible for activity. Moreover, the presence of PfApicortin needed to be confirmed experimentally in the blood stage of the parasite. Stage-specific semiquantitative PCR and western blotting validated the expression of PfApicortin in ring, trophozoite and schizont stages (Fig. 3). Furthermore, confocal microscopy of immunostained parasites proved the abundance of PfApicortin in schizont and merozoite stages. PfApicortin was also found to colocalize with MTIP: the high colocalization coefficient indicates a possible interaction of PfApicortin with the parasite cytoskeletal protein complex during invasion (Fig. 4). Accumulation of PfApicortin at the apical end of merozoites also supports its putative role in apical complex formation, as reported in another apicomplexan parasite *Toxoplasma gondii* (Nagayasu et al., 2017).

Parasite growth was retarded in erythrocytes loaded with miRNA mimic. Further evaluation revealed that PfApicortin expression was reduced in miRNA-loaded erythrocytes, as suggested by western blot analysis and immunofluorescence assay. The rate of invasion decreased significantly in erythrocytes loaded with miRNA mimic 72 h after setting up the assay, indicating a possible role of PfApicortin in invasion of the parasite (Fig. 6). Among the candidate molecules, miR-197-5p was found to be more potent than miR-150-3p for anti-apicortin activity, as suggested by the micronemal secretion data (Fig. 6D, Fig. S3B). However, miR-150-loaded erythrocytes led to growth inhibition and reduced invasion of parasites upon infection (Figs 5B,C,E and 6A,C). The possible reason behind this phenomenon can be explained with reference to the translocation and intra-erythrocytic stability of miRNAs. Quantitative PCR (qPCR) using miRNA-specific primers showed a difference in levels of the two miRNAs at different time points after invasion. The level of miR-150 was approximately 40-fold (Fig. 7A, *P*<0.01) over the control at all three time points, whereas the level of miR197 was higher: 80-fold (*P*<0.001), 100-fold (*P*<0.01) and 50-fold (*P*<0.01) at 24 h, 48 h and 72 h post-infection, respectively (Fig. 7A). Therefore, the translocation efficiency of miR-197 might be higher than for miR-150, which also supports its higher anti-apicortin activity and effect on micronemal secretion. Thus, miR-197 seems a promising candidate for nucleotide-based antimalarial therapeutics.

### Conclusion

In summary, our data confirm the role of host erythrocyte miRNA-mediated downregulation of malaria parasite gene expression. In this regard, we identified two miRNAs, miR-197-5p and miR-150-3p, which affect parasite growth and invasion when enriched in erythrocytes. We also identified a novel parasite target protein, PfApicortin, which has a possible role in growth and invasion in terms of stabilizing cytoskeletal assembly of the parasite. The mechanism of inhibition might be through translation repression of protein synthesis, owing to the formation of a mRNA-miRNA hybrid (LaMonte et al., 2012; Wang et al., 2017; Dandewad et al., 2019) (Fig. 7C). Further experiments will enable us to determine the interaction of PfApicortin with other parasite proteins.

## MATERIALS AND METHODS

### *In silico* hybridization of parasite genes with erythrocytic miRNA

MicroRNA sequences found in mature erythrocytes were selected and hybridized with *P. falciparum* and *P. vivax* transcripts primarily expressed in the trophozoite and schizont stages. *P. falciparum* and *P. vivax* mRNA sequences were downloaded from PlasmoDB (https://plasmodb.org/plasmo/) and miRNA sequences were taken from miRBase (http://www.mirbase.org/). Hybridization was carried out using RNAhybrid (Rehmsmeier et al., 2004). MicroRNA sequences were hybridized in domain mode to find the most favourable site for binding of a short nucleotide sequence onto a longer target sequence on the basis of minimum free energy of binding. The threshold for binding energy was set at −20 kcal/mol and *P*-value approximation was kept flexible. The mRNA-miRNA pairs were plotted according to the increasing order of minimum free energy for binding.

### Tissue culture and transfection

HEK 293T cells (obtained from ATCC CRL-3216) were cultured in T25 flasks (Nunc™) using DMEM high glucose (Thermo Fisher Scientific, MA) supplemented with 10% foetal bovine serum (Invitrogen, Carlsbad, CA, USA), 2 g/l sodium bicarbonate (Himedia, India) and 1% antibiotic-antimycotic solution (Thermo Fisher Scientific, MA, USA). Cells were incubated in a humidified chamber at a temperature of 37°C and 5% CO_2_ (Thermo Fisher Scientific). Cells were seeded in six-well plates with a density of 5×10^5^ cells per well and transfection was performed using Lipofectamine 2000™ (Invitrogen), according to the manufacturer's protocol. Briefly, 1 μg of target gene containing plasmid and 2 μg of miRNA-encoding plasmid were mixed with 2.5 μl of Lipofectamine in OptiMEM (Invitrogen) and incubated for 30 min at room temperature for complex formation. The mixture was added to the cells, incubated at 37°C for 4 h and complemented with complete DMEM followed by further incubation at 37°C, 5% CO_2_ for 48 h.

### Parasite culture and invasion assay

*P. falciparum* strain 3D7 (obtained from BEI resources MRA-102) was cultured using O+ packed erythrocytes together with complete RPMI 1640 supplemented with AlbumaxII (Gibco, CA, USA), hypoxanthine (Sigma-Aldrich, MA, USA) and gentamycin (Gibco) at 37°C with a mixed gas composition of 2% O_2_, 5.5% CO_2_ and 92.5% N_2_. The invasion assay was set up according to previously published methods. Briefly, late trophozoites or schizonts were purified from mixed parasite culture using 65% Percoll (GE Healthcare) and washed with incomplete RPMI. Erythrocytes (both normal and those loaded with miRNA mimic) were seeded in a 96-well plate (Nunc, Thermo Fisher Scientific) with 4% hematocrit and invasion was set up at 1% parasitemia. Growth of the parasite was monitored at different time points by making erythrocyte smears and staining parasites with Giemsa stain (Sigma-Aldrich).

### Treatment of parasites with miRNA mimics

*P. falciparum* 3D7 was grown using the above-mentioned conditions. Mature schizonts (40-42 h old) were incubated with 10 μM miR-150-3p, miR-197-5p or scrambled nucleotide (*mir*Vana^®^ miRNA mimic, Thermo Fisher Scientific) as negative control. After 8 h incubation, the invasion assay was set up with treated schizonts and fresh O+ packed erythrocytes with 4% hematocrit at 1% parasitemia. Merozoites were also collected from the treated parasites and used to prepare cell lysate for western blotting.

### Enrichment of erythrocytes with miRNA mimics

Erythrocytes were loaded with miRNA mimics using methods published previously (Chandramohanadas et al., 2009; Murphy et al., 2006; Govindarajalu et al., 2019). Briefly, erythrocytes were washed with phosphate-buffered saline (PBS) then resuspended in lysis buffer (5 mM K_2_HPO_4_, 1 mM ATP, pH 7.4) that also contained miRNA mimic at a concentration of 10 μM as cargo; erythrocytes were then incubated for 1 h at 4°C. The gene encoding PfApicortin was amplified from *P. falciparum* cDNA and 2 μg of the amplified product was loaded in erythrocytes as DNA control. Scrambled miRNA mimic and anti-miR-197-5p (*mir*Vana^®^ miRNA mimic, Thermo Fisher Scientific) were also loaded as negative controls. Cells were resealed using resealing buffer (475 mM KOAc, 25 mM Na_2_HPO_4_, 25 mM MgCl_2_, 237.5 mM KCl, pH 7.5) for 1 h at 37°C followed by washing with incomplete RPMI and storage at 4°C for further experiments (reagents used were from Sigma-Aldrich).

### Preparation of plasmid constructs, protein expression and antibody generation

Pre-miR-150 and pre-miR-197 were cloned in miRNA cloning and expression vector miRNASELECT™ pEP-Mir using BamH1-NheI sites (primers listed in Table S2). The gene encoding PfApicortin was cloned in SnaBI-digested and dephosphorylated plasmids pCMV (mammalian expression) and pET (bacterial expression). The cloned construct (pET-PfApicortin) was transformed in *Escherichia* *coli* codon plus strain and PfApicortin was expressed under the conditions: induction with 1 mM IPTG (Sigma-Aldrich) followed by incubation at 37°C for 6 h with shaking at 150 rpm. Recombinant protein was purified by Ni-NTA (Qiagen, Hilden, Germany) chromatography followed by elution with 250 mM imidazole (Sigma-Aldrich). The purified protein was injected into BALB/c mice (6 weeks, female), which were bled twice following the first and second booster. Antibodies against MSP1, MTIP and AMA1 were generated in-house. Animal handling and sera generation were performed according to the Committee for the Purpose of Control and Supervision of Experiments on Animals (CPCSEA) guidelines and approved by the Institutional Animal Ethics Committee (IAEC), Jawaharlal Nehru University, New Delhi. All experiments were carried out in accordance with the guidelines and regulations of Jawaharlal Nehru University and approved by institutional IBSC committee.

### RNA isolation, poly(A) tailing of miRNAs and qPCR

Total RNA was isolated from transfected cells and parasites using Tri reagent (Life Technologies, CA, USA) following the manufacturer's protocol. RNA was quantified by nanodrop (Thermo Fisher Scientific) and cDNA was synthesized using the High capacity cDNA Synthesis Kit (Applied Biosystems) according to the manufacturer's protocol with 1 μg of total RNA per reaction. In order to synthesize cDNA from mature miRNAs, poly(A) tailing of the parasite total RNA was performed using poly(A) polymerase (NEB, MA, USA) according to the manufacturer's protocol before cDNA synthesis. Semiquantitative PCR was set up for checking apicortin expression along with control genes using the conditions described: denaturation at 95°C for 1 min, annealing at 54°C for 30 s followed by extension at 64°C for 1 min with 25 cycles and preceded an initial denaturation at 95°C for 5 min. Extension of mammalian GAPDH was performed at 72°C. The PCR product was run on a 2% agarose gel (SRL chemical, India) and the image captured using gel documentation system (Bio-Rad, CA, USA). qPCR was set up using Power Up SYBR Green™ Master Mix (Applied Biosystems, CA) with primers designed for PfApicortin and human GAPDH. For the detection of miRNAs, polyadenylated parasite cDNA samples were probed with primers designed for miR-150-3p and miR-197-5p using miRPrimer software (https://sourceforge.net/projects/mirprimer/; Cirera and Busk, 2014) together with Pf18S primer. Reactions were set up in triplicate in Step-One Plus Real-Time PCR instrument (Applied Biosystems) using the following conditions: initial denaturation at 95°C for 5 min, annealing at 60°C for 30 s and extension at 72°C for 1 min with 40 cycles. Fluorescence was measured after extension and melt curve analysis was also added. Data were analysed using Step-One software (Applied Biosystems).

### Western blotting

Transfected cells were lysed in RIPA lysis buffer (G Biosciences, India) followed by centrifugation at 15,600 ***g*** at 4°C for 15 min. Supernatant was collected and stored at −20°C after adding 1×protease inhibitor cocktail (Thermo Fisher Scientific). Parasite lysate was prepared by lysis of erythrocytes with 0.1% saponin (Sigma-Aldrich) and suspension of parasite in RIPA lysis buffer followed by sonication (Vibra Sonics) with an amplitude of 30 and the conditions described: running for 20 s followed by a break of 30 s repeated for 3 min. The suspension was centrifuged at 15,600 ***g*** for 10 min and stored at −20°C after addition of 1× Protease Inhibitor Cocktail (Sigma-Aldrich) and 1 mM phenylmethylsulphonyl fluoride (PMSF, Sigma-Aldrich). Cell and parasite lysates were separated using 12% SDS-PAGE, following protein quantification with the Bicinchoninic acid assay kit (G Biosciences, India). Proteins were transferred to a nitrocellulose membrane (Bio-Rad) and blocked overnight at 4°C with 5% skim milk (Himedia, India). The blot was incubated with anti-apicortin primary antibody (1:1000) for 2 h at room temperature, followed by washing with PBS and incubation with horseradish peroxidase-conjugated antimouse secondary antibody (1:5000, Sigma-Aldrich) for 1 h at room temperature. Antibody binding was probed using an Enhanced Chemiluminescence Kit (Bio-Rad). The same protocol was followed for immunoblotting of AMA1 (1:500) and GAPDH (1:5000; Thermo Fisher Scientific). Band intensity was quantified using ImageJ software (NIH).

### Immunofluorescence assay

HEK 293T cells were seeded on sterile coverslip before transfection. Fixation of the transfected cells was achieved by incubation in chilled methanol for 15 min. For parasites, smears of infected erythrocytes were prepared and fixation was carried out using the same procedure. Cells/smears were blocked overnight at 4°C with 3% bovine serum albumin (Sigma-Aldrich) followed by incubation with primary antibody for 2 h at room temperature. After washing, incubation with secondary antibody (dilution 1:300; conjugated with Alexa Fluor 488/546, Invitrogen) was performed for 1 h at room temperature. Cells/smears were mounted using DAPI-antifade (Invitrogen) and images were captured using a confocal microscope at 100× magnification (Olympus Corporation, Tokyo, Japan). Analysis of the images was performed using NIS Elements software.

### Statistical analysis

*P*-values were calculated applying two-tailed Student's *t*-test wherever required. Results are represented as mean±s.d. for three independent experiments.

## Supplementary Material

Supplementary information
