# Supplementary material for: Targeted repression of Plasmodium apicortin by host microRNA impairs malaria parasite growth and invasion
Source: Dis Model Mech. 2020 Jun 3;13(6):dmm042820. doi: 10.1242/dmm.042820 (PMC7286292; doi:10.1242/dmm.042820)
Supplement: Supplementary information [file dmm-13-042820-s1.pdf]

FigS1

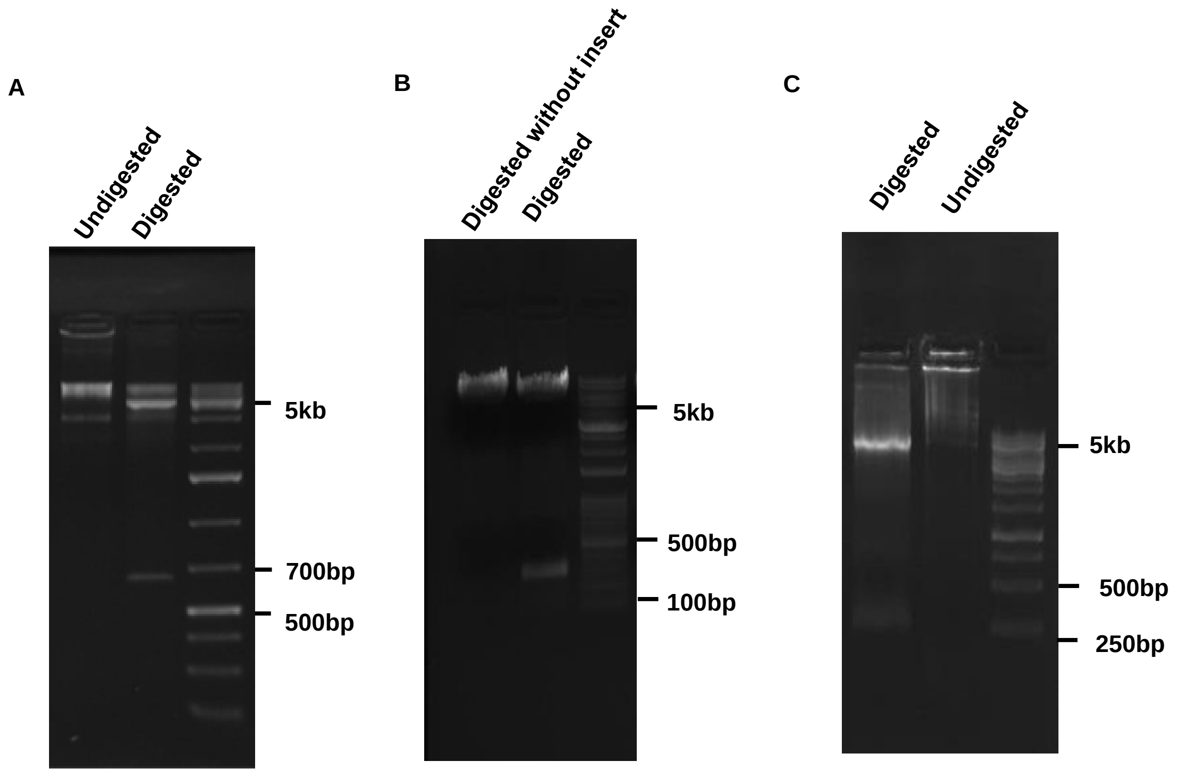

Fig. S1. Preparation of Constructs. (A) Restriction digestion of pCMV vector showing insert of apicortin, **(B)** Restriction digestion of pEPMiR with BamHI and NheI showing insert of Pre-miR150 (274bp),(**C**) Restriction digestion of pEPMir with BamHI and NheI showing insert of Pre-miR197 (275bp).

Disease Models & Mechanisms • Supplementary information

FigS2

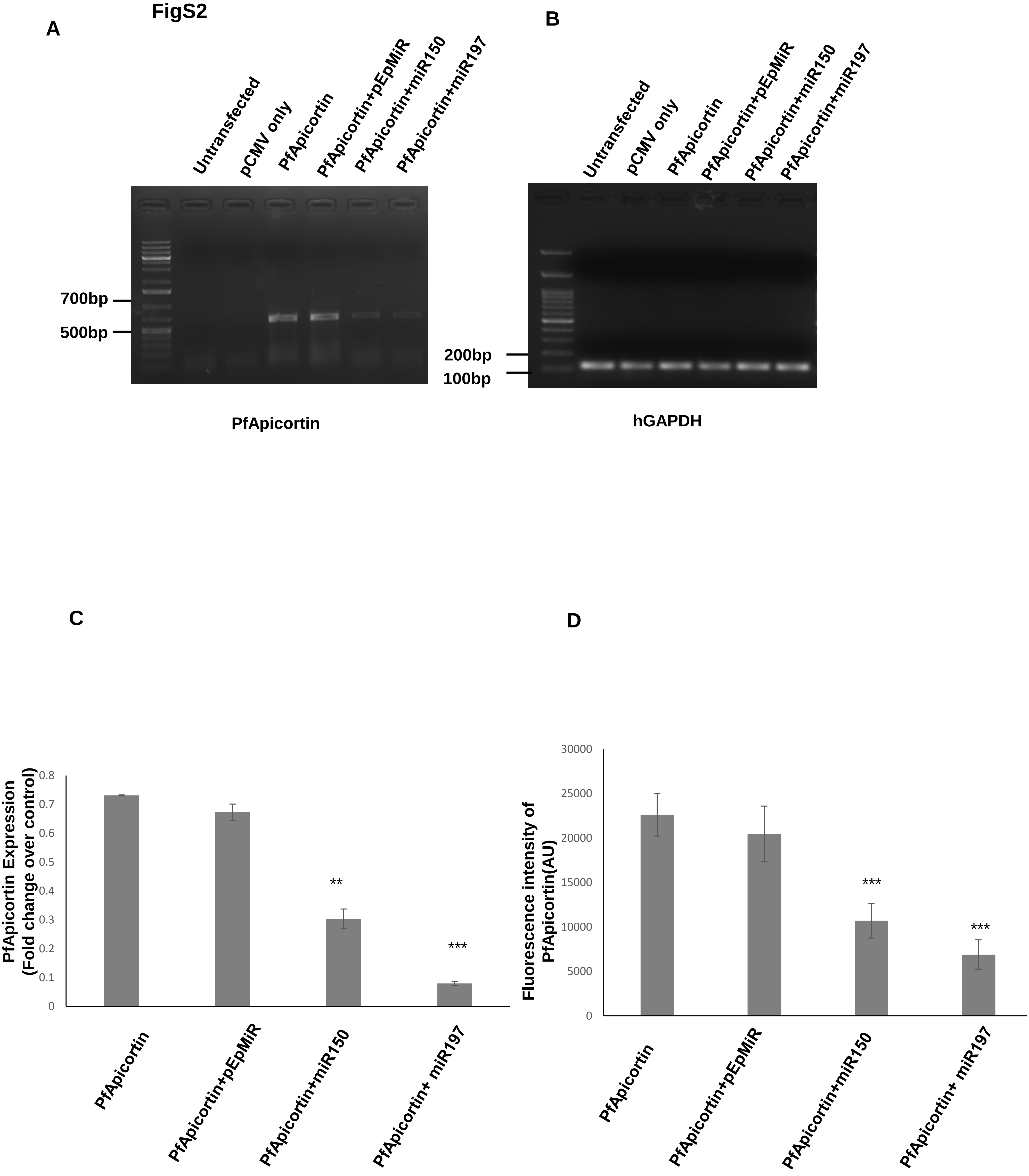

Disease Models & Mechanisms • Supplementary information

Fig. S2. Full agarose gel and quantitation of protein expression shown in fig. 2A, 2C & 2D. Agarose gel image of PfApicortin and hGAPDH expression in HEK293T cells cotransfected with miR150 and miR197 (Fig. 2A), **(C)** Fold change in expression of PfApicortin over control in HEK293T cells cotransfected with miR-150 and miR-197(Fig. 2C), **(D)** Graph showing fold change in fluorescence intensity of PfApicortin miRNA co transfected cells (miR150, *** $p<0.001$; miR197, *** $p<0.001$).

FigS3

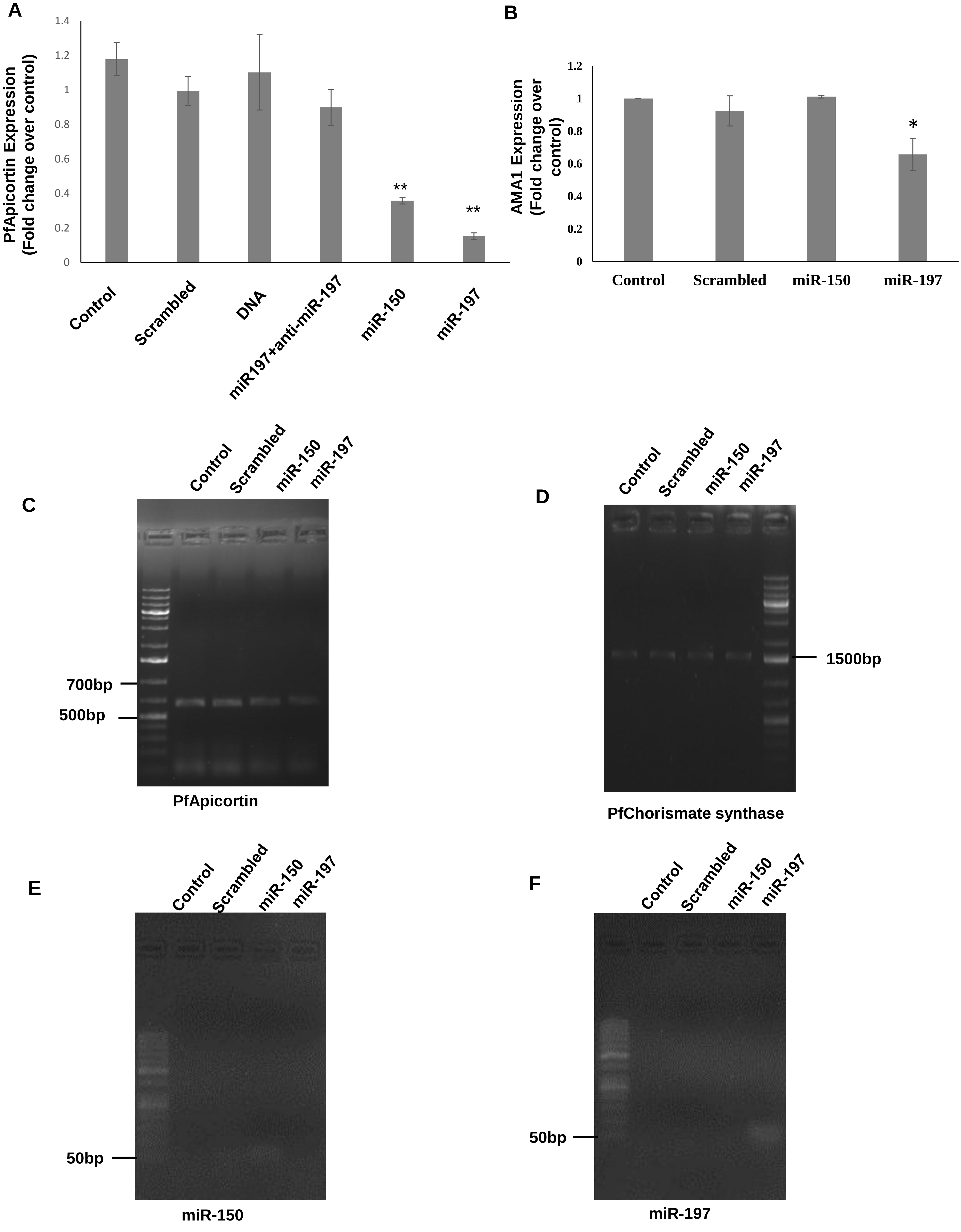

Disease Models & Mechanisms • Supplementary information

Fig. S3. Level of expression of PfApicortin, chorismate synthase and AMA1 in miRNA mimic treated merozoites along with miRNA mimic translocation in parasite. (A) Graph showing fold change in PfApicortin expression (Fig. 5C; miR150, **$p<0.01$; miR197, **$p<0.01$), **(B)** Graph showing fold change in AMA1 expression over control (Fig. 6D, miR 197, * $p<0.05$), **(C)** Full gel images showing PfApicortin and chorismate synthase expression in parasite infecting miRNA mimic loaded erythrocytes (Fig. 5E), **(D)** Presence of miR150 in parasite infecting miR150 mimic loaded erythrocytes, **(E)** Presence of miR197 in parasite infecting miR197 mimic loaded erythrocytes.

Disease Models & Mechanisms • Supplementary information

Table S1. List of microRNAs selected for *in silico* hybridization

miRNA (Column1)	miRNA (Column2)
hsa-miR-16	hsa-miR-181a
hsa-miR-223	hsa-miR-222
hsa-miR-150	hsa-miR-324
hsa-miR-96	hsa-miR-196a
hsa-miR-15a	hsa-miR-106a
hsa-miR-124	hsa-miR-106b
miR-144	hsa-miR-142
Let-7b	hsa-miR-144
Let-7d	hsa-miR-148
Let-7g	hsa-miR-126
miR-93	hsa-miR-191
miR-98	hsa-miR-19b
miR-181b	hsa-miR-20a

miRNA (Column3)	miRNA (Column4)
hsa-miR-20b	hsa-26a
hsa-miR-21	hsa-miR-15b
hsa-miR-25	hsa-miR-594
hsa-miR-26b	hsa-miR-191
hsa-miR-29c	hsa-miR-30c
hsa-miR-30b	hsa-miR-27a
hsa-miR-342	hsa-miR-182
hsa-miR-374a	hsa-miR-140
hsa-miR-92	hsa-miR-21
hsa-miR-486	hsa-miR-101
hsa-miR-451	hsa-miR-320
hsa-miR-24	hsa-miR-197
hsa-miR-126	

Disease Models & Mechanisms • Supplementary information

Table S2. List of primers used for cloning, semi quantitative and quantitative PCR. Bold nucleotides indicate restriction sites.

Primers	Sequences
pET_Apicortin	ACGAATTC**TACGTA**ATAAATTCTTGTAAGCATCCATGC (F)<br>ACGAATTC**TACGTA**AGTTAGAAAGTGAAGACTCAAG (R)
pCMV_ Apicortin	ACGAATTC**TACGTA**ATAAATTCTTGTAAGCATCCATGC (F)<br>ACGAATTC**TACGTA**AGTTAGAAAGTGAAGACTCAAG (R)
miR150_pEPMir	ATA**GGATCC**CCCCCGCCCTCCCTGGACCTGGGTA (F)<br>ATT**GCTAGC**AAGGGGAGAGACGCATAAAAGCC (R)
miR197_pEPMir	TCGA**GGATCC**AAATGTTTTCATGCTTTTACCTAGC (F)<br>TCGA**GCTAGC**TAGTCCAAAACAGAGTATTCCT (R)
Apicortin_RT	CATCCATGCTGTGAGGAAGATA (F)<br>GGAGCATGTTCATTCCTTTCTTT (R)
18s	CCGCCCGTCGCTCCTACCG (F)<br>CCTTGTTACGACTTCTCCTTCC (R)
EBA-175	AATTTCTGTAAAATATTGTG ACCATATG (F)<br>GATACTGCACAACACAGATTTCTTG (R)
Intron_Specific	GACTTCCACCTTATATTCCATG (F)<br>TATAAGCCGTAGTTTTATCCCTA (R)
hGAPDH	GACCACTTTGTCAAGCTCATTTC (F)<br>CTCTCTTCCTCTTGTGCTCTT (R)
miR-150-3p_RT	CAGCTGGTACAGGCCT (F)<br>CCAGTTTTTTTTTTTTTTTTCTGTCC (R)
miR-197-5p_RT	GGGTAGAGAGGGCAGTG (F)<br>TCCAGTTTTTTTTTTTTTTTTCCTCC (R)

Disease Models & Mechanisms • Supplementary information
